# Zirconia-Based Ultra-Thin Compact Flexible CPW-Fed Slot Antenna for IoT

**DOI:** 10.3390/s19143134

**Published:** 2019-07-16

**Authors:** María Elena de Cos Gómez, Humberto Fernández Álvarez, Blas Puerto Valcarce, Cebrián García González, John Olenick, Fernando Las-Heras Andrés

**Affiliations:** 1 TSC Electrical Engineering Department, University of Oviedo, 33203 Gijón, Spain; 2 IDONIAL Centro Tecnológico, 33203 Gijón, Spain; 3 ENrG Inc., Buffalo, NY 14207, USA

**Keywords:** flexible antenna, compact antenna, ultra-thin antenna, ceramic antenna, antenna for IoT, electrotextile antenna, inkjet-printed antenna

## Abstract

An ultra-thin compact flexible CPW-fed slot monopole antenna suitable for the Internet of Things (IoT) applications was achieved as a result of exploring the use of Zirconia-based ENrG’s Thin E-Strate^®^ for the antenna’s design. The electromagnetic characterization of the novel material at the frequency range of interest was analyzed. A comparison was made concerning the required dimensions and the simulation results regarding impedance matching and radiation properties, for three different dielectric substrates: Novel flexible ceramic (ENrG’s Thin E-Strate), rigid Arlon 25N, and flexible Polypropylene (PP). Two different metallization techniques—electrotextile-based and inkjet printing—were used in the fabrication of prototypes based on ENrG’s Thin E-Strate. Return losses measured results for the fabricated prototypes with both procedures was compared, as well as with simulation. The best prototype on the ENrG’s Thin E-Strate was compared with one on Arlon 25N, in terms of radiation properties in an anechoic chamber, and conclusions were drawn.

## 1. Introduction

The Internet of Things (IoT) refers to objects (specific and everyday ones) that can be connected through Internet networks due to the embedding of electronics. Therefore, IoT is the migration of the Internet beyond people. The devices can communicate and interact with others over the Internet, and they can be remotely monitored and controlled.

IoT has not fully penetrated consumers’ daily life with wearable devices yet. Three main factors affect the lagging status of consumer IoT technology: Feasibility, reliability of the connection between devices, and functionality of devices. However, wearable technology is already being developed and has a bright future in healthcare with the passive monitoring of vital statistics. The wearables market is still in the early phases of expansion, and currently dominated by health, wellness, and activity tracking devices—despite industry developments pointing to an increasing number of use cases. The wearables industry is thus involved in finding the use cases that will drive mass adoption by the consumers. Furthermore, wearable devices are, nowadays, at the heart of just about every discussion related to the IoT, and the full range of new capabilities which pervasive connectivity can bring.

Wearable electronics (WE), by virtue of an IP address, join the IoT and, as an additional advantage, do not have to exist as standalone devices. People everywhere will be able to interact with things by using WE [1,2,3] and the IoT.

During the last decade we have witnessed a great development concerning flexible and/or miniaturized electronic devices for facilitating their integration in handheld equipment and/or in clothing. The advances regarding the design and fabrication of antennas [4,5,6,7,8,9] based on new materials [10,11,12,13,14] and the use of innovative manufacturing techniques [14,15,16,17,18,19,20] are key steps towards these WE.

As previously mentioned, a large market is foreseen in the near future for devices and emerging technologies with applications in 5G and IoT. For these new standard definitions, a wide variety of frequency bands are considered, both below 6GHz and at much upper frequencies (such as 24 GHz, 28–29 GHz, 60 GHz, and even higher values), with emphasis on ISM bands. With regard to the IoT, the preferred frequencies traditionally allocated for WLAN (around 2.4 GHz, 3.6 GHz, 4.9 GHz, 5 GHz, 5.9 GHz) and wireless sensor network communications (ZigBee, Bluetooth, RFID, NFC,….) are mostly around 2.45 GHz [10,11,21,22]. The reasons behind such preferences are the lower losses and the know-how, concerning not only the channel characterization, but also the previously developed electronics. Furthermore, frequencies below 1 GHz [23]—such as 900 MHz and 700 MHz—are also considered for some 5G applications, although frequency re-farming is still pending in some countries. In view of these perspectives, the design of flexible and/or miniaturized antennas suitable for integration in next-generation devices is of great interest, although challenging.

Wearable electronics (WE) are enabled by the miniaturization and integration of components. Cameras, sensors, speakers, computer chips, and other components continue getting smaller while becoming more capable. The same applies to the antennas, as key parts of wearable communication systems. However, the textile dielectrics generally used in WE have a low relative dielectric permittivity ε_r_ (ranging from 1.17 of fleece to 2.95 of leather) [15], which does not allow for reducing the size of the antennas at frequencies below 6GHz. Other non-textile employed materials, such as polystyrene foam, exhibit even lower values (ε_r_ = 1.02); the only one reporting a higher value is neoprene rubber (ε_r_ = 5.2), which is used, for example, in garments for watersports. The development and use of novel flexible dielectrics, with much higher relative dielectric permittivity than textiles and the mentioned ones for WE, needs to be explored. However, maintaining the flexibility while increasing the relative dielectric permittivity is a great challenge.

This work aims to design and manufacture an ultra-thin, flexible antenna based on a novel ceramic material, ENrG’s Thin E-Strate^®^, for IoT applications at frequencies around 2.7 GHz and 5.8 GHz (covering several of the intended bands), and compare its dimensions and performance (concerning matching and radiation properties) with the ones obtained using conventional rigid Arlon 25N dielectric and flexible polypropylene substrates.

This contribution is organized as follows: First the characteristics of the novel flexible ceramic material are described, as well as the ones of the conventional Arlon 25N and polypropylene. Then, the CPW-fed antenna design based on the three materials is presented and the obtained results are compared based on simulation. Prototypes of the designed antenna are fabricated using two different metallization techniques and their performance is compared with the simulation in terms of return losses. The best prototype of the antenna obtained on ENrG’s Thin E-Strate is then compared with a prototype on Arlon 25N, based on radiation properties obtained through measurements in an anechoic chamber. Finally, some conclusions are drawn.

## 2. Dielectric Substrates for the Design of the Antenna

In this section, the novel flexible ceramic material under evaluation for antenna design is presented, along with two conventional ones already used for such purpose: Flexible polypropylene (PP), and Arlon 25N which is rigid. Moreover, the ceramic material is electromagnetically characterized, since these data are crucial for the design purpose. In addition, other relevant properties of this dielectric (mechanical, thermal, chemical…) is provided.

### 2.1. Ultra-Thin Flexible Ceramic Material

ENrG’s Thin E-Strate^®^ [24] is an ultra-thin, flexible, ceramic substrate which has properties for developing higher performance products than those based on traditionally available materials. The most remarkable properties are: Flexibility, mechanically robust, light-weight ceramic; high temperature tolerance; high thermal shock tolerance; impermeable to gases and moisture; chemically inert (in most harsh chemical environments); easily coated with conductive metals; and high wear and abrasion resistances. It is available in sheet, wafer, or ribbon formats with thicknesses of 20 and 40 μm.

From an electromagnetic point of view, ENrG’s Thin E-Strate is a Zirconia-based ceramic that exhibits a high relative dielectric permittivity and a reduced loss tangent (well below 0.01). Preliminary studies yielded relative dielectric permittivity values of ε_r_ = 26 at 100 KHz and ε_r_ = 28 at 10 GHz. However, it is important to take into account that the latter characterization value was obtained for a 20-μm-thick sample using a Keysight Technologies 85072A 10 GHz split-cylinder resonator. Measuring such a thin sample with this equipment is not very accurate, due to small disturbance of the inherent cylinder resonance by the sample, and such disturbance is the base of the characterization. In fact, it is recommended by the fabricator using samples in the 0.05–5mm range and typically 1 mm. Subsequent characterization using a 40-μm-thick sample (the one available closer to the recommended thickness) yielded ε_r_ = 22.14 at 10GHz with a loss tangent tanδ = 0.0012. Measurements conducted with split-post dielectric resonator at lower frequencies rendered ε_r_ = 22.19 and tanδ = 0.0009 at 3 GHz, and ε_r_ = 22.02 and tanδ = 0.0011 at 1 GHz.

All these methods are useful for electromagnetically characterizing the material when it is taken as a pure sample for being used alone. However, the values obtained through this kind of analysis, although very useful, could lead to shifts in frequency and variations in the bandwidths when used in simulation for the antenna (or any type of microwave device) design. The reason is that these methods are not based on utilizing the transmission lines which are actually used to feed and/or shape the device. Depending on the fabrication procedure and the dielectric under consideration’s properties, the transmission lines will suffer under-etching or over-etching (becoming narrower or wider than due), thus resulting in frequency shifts. For example, it is well known that although the RO3010™ yields ε_r_ = 10.2 at 10 GHz, the own manufacturer Rogers Corp. recommends using 11.2 for design [25]. The same applies for many commercially available dielectrics.

**Figure 1:**
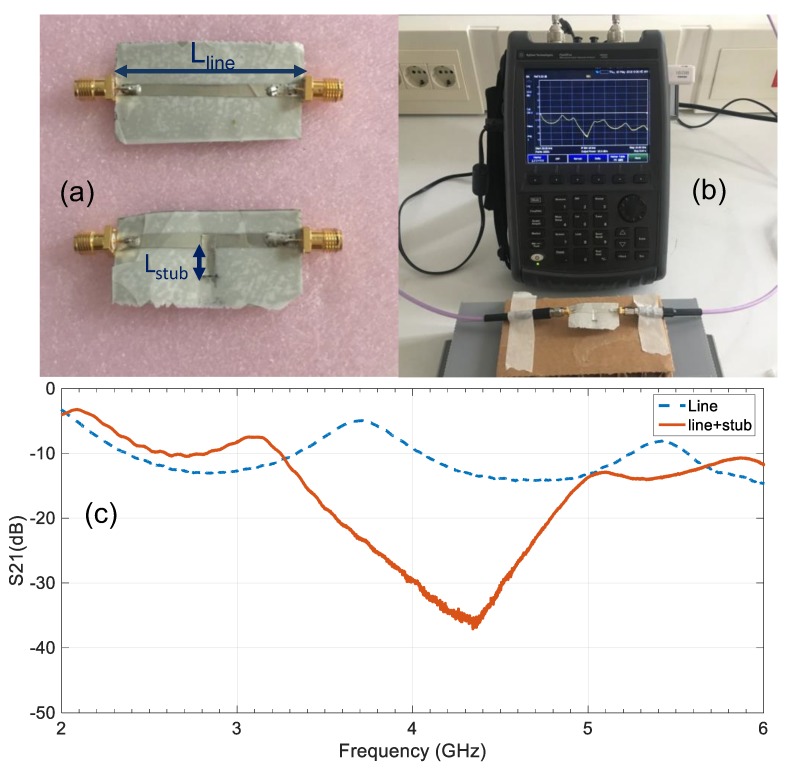
Electromagnetic characterization of the ENrG’s Thin E-Strate at 4 GHz: (**a**) Prototypes of the microstrip line and microstrip T-resonator; (**b**) measurement set-up for the S-parameters; (**c**) measurement results for the S21 parameter.

Taking this into consideration, further characterization tasks were conducted using a microstrip line and a T-resonator [26] on the ENrG’s Thin E-Strate as base. These structures were fabricated, and subsequently, their S21 parameter was simulated using HFSS, tuning ε_r_ and tanδ in simulation to match the measurement results. Generally, a 50 Ω impedance is chosen for the microstrip line to avoid mismatches with the vector network analyzer (VNA) used for measurements. However, for a high relative permittivity dielectric, such line would be too narrow to be manufactured with conventional techniques. A 2.5 mm wide line was used for simple fabrication instead, which corresponds to very low impedance at the frequencies under study (less than 5 Ω). The mismatch with the VNA lead to ripple in the S21, but the method remained valid since this happened both in simulation and measurement. The T-resonator is a line of identical characteristic impedance with a λ_g_/4 stub at the intended characterization frequency. In this case, 4 GHz was chosen as it is in between the two operating frequencies of the antenna to be designed. The fabricated samples with 40 microns thickness are shown in Figure 1a, whereas the measurement set-up with the T-resonator sample appears in Figure 1b. The obtained results for both samples are represented in Figure 1c, where it is clearly visible the resonance of the T-resonator around 4 GHz. The simulations conducted with HFSS to obtain such results required using ε_r_ = 22, tanδ = 0.001 and h = 0.04 mm thickness. These results make sense since they are close to the ones rendered by the previously mentioned methods for identical sample thickness, as can be observed in Table 1. Furthermore, as the antenna to be designed will be fed by a CPW line, a simple CPW-fed monopole antenna (as the one already used by the authors with other materials [27]) was designed and tested using the later retrieved values ε_r_ = 22, tanδ = 0.001. Very good agreement was found, so that it can be considered that those values are the proper ones to be considered for design at this frequency range.

According to the available technical information [28], Thin E-Strate can offer new options in the development of harsh environment sensors, power electronics circuit boards, LEDs and luminaires, contoured circuit boards for space and aviation, micro-batteries, and thin-film photovoltaic cells, to name a few potential applications. Thus, it opens the path to smaller, lighter products. In view of this, it seems very interesting to explore the possibility of using this novel flexible ceramic material for antennas.

### 2.2. Alternative Conventional Materials

As mentioned in the introduction, two dielectric substrates more conventionally used for antenna fabrication are considered for the IoT antenna design.

One is rigid, the Arlon 25N with relative dielectric permittivity ε_r_ = 3.38, loss tangent tanδ = 0.0025, and thickness h = 0.812 mm. (This material could also be replaced by Rogers 4003C with very similar electromagnetic characteristics while using the same thickness.)

The other is flexible polypropylene (PP), with ε_r_ = 2.26, loss tangent tanδ = 0.002, and thickness h = 0.45 mm—which has been previously characterized and used for antenna fabrication at 2.45 GHz and 5.8 GHz frequencies by members of this research team [27], and also used by other authors.

## 3. CPW-Fed Slot Monopole Antenna Design

Coplanar-Waveguide-feeding (CPW-feeding) has been chosen, since it provides much wider bandwidth than microstrip feeding, while it requires metallizing only one layer [27]—which is cheaper and easier to fabricate. The reference impedance is 50 Ω.

### 3.1. Antenna Geometry and Optimized Dimensions

For the design of the feeding line, with a width W_L_ and gap g, not only the 50 Ω impedance but also the dimensions of commercially available connectors have been taken into account.

**Figure 2:**
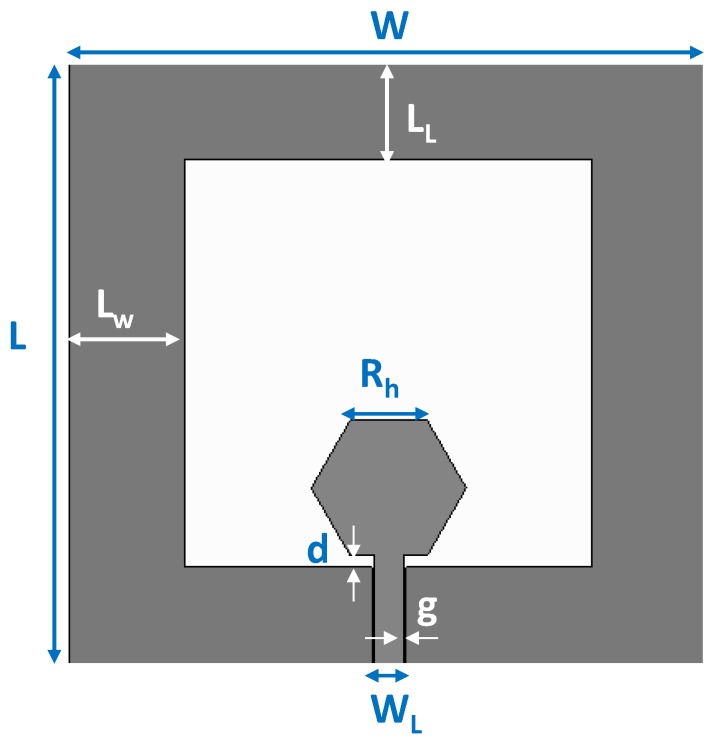
Geometry of the CPW-fed slot monopole antenna.

Figure 2 shows the geometry of the monopole antenna. The radiating slot is defined by surrounding a hexagonal-shaped patch, arising from the CPW-feeding line, with metallic strips connected to both sides of the ground plane.

The dimensions of the antenna have been optimized through FEM-based 3D electromagnetic simulation using HFSS commercial software, so that it is properly matched at the intended IoT bands (around 2.75 GHz and 5.8 GHz) for the three dielectric substrates (ENrG’s Thin E-Strate, Arlon 25N, and polypropylene (PP)). The aim is not so much to design the best possible antenna, but to design a compact antenna that properly covers the intended bands—and to compare the size and performance with the three materials to study the possibilities of the novel ceramic substrate for the antenna’s applications. The dimensions obtained for the optimized antenna design, in terms of impedance matching, based on the different materials are detailed in Table 2.

A parametric analysis was conducted to optimize the design of the antenna at the intended frequencies of operation for IoT. Increasing W improves the matching in the upper frequency band whereas increasing L does it in the lower band while shifting-down the upper resonance. A higher value of R_h_ decreases the upper resonance frequency, though if its value is too low there is only one resonance in the intended frequency range. A higher d value improves the matching, especially for the upper band. L_W_ mainly influences the matching of the lower band, whereas L_L_ varies the matching while shifting both frequency bands so that a trade-off has to be adopted.

According to Table 2, the total size of the optimized CPW-fed slot monopole antenna based on ENrG’s Thin E-Strate is 40.92 cm^2^, whereas for both Arlon 25N and PP is 48.3 cm^2^. Thus, using the novel Thin E-Strate substrate lead to a 15.28% size reduction. Furthermore, this new substrate is 20 and 11.25 times thinner than Arlon 25N and PP, respectively. Therefore, the antenna on the novel flexible substrate is not only smaller than on the rigid Arlon 25N and the flexible PP, but also much thinner.

### 3.2. Antenna Matching

**Figure 3:**
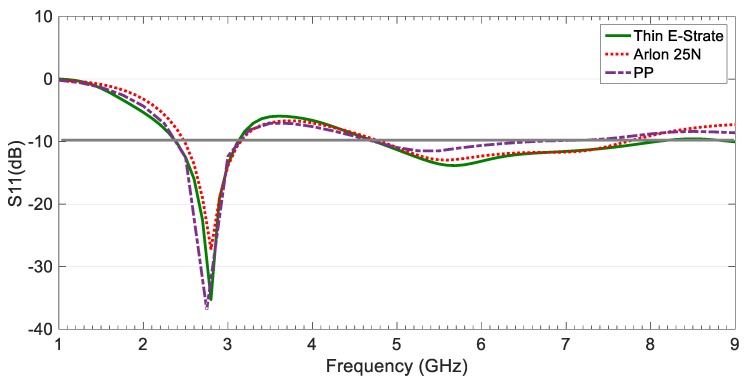
Return loss simulation results for the CPW-fed slot monopole antenna based on ENrG’s Thin E-Strate, Arlon 25N, and polypropylene (PP) substrates.

From the return loss simulation results shown in Figure 3, it can be observed that the CPW-fed slot monopole antenna is well matched at the intended IoT bands for the three dielectric substrates under consideration, and for the corresponding dimensions included in Table 2.

The flexible PP provides better impedance matching than the Arlon 25 N for the lower frequency band around 2.7 GHz, whereas for the upper band around 5.8 GHz, the Arlon 25N renders better matching than the PP. Moreover, the Thin E-Strate allows very good impedance matching for both frequency bands. The antenna based on ENrG’s Thin E-Strate is better-matched than one based on the Arlon 25N for both bands, is at a similar level than for the design based on the PP for the lower band, and much better for the upper band.

The specific operation frequencies and bandwidths obtained in simulation for the CPW-fed slot monopole antenna based on the three dielectrics are indicated in Table 3. It can be observed that the use of the novel ENrG’s Thin E-Strate makes it possible to obtain the same bandwidth for the lower band than with the PP (27% vs. 23% obtained for the Arlon 25N) and the widest bandwidth for the upper band (51% vs. 47% for the Arlon 25N and 33% for the PP).

These results are very remarkable, taking into consideration that the flexible ENrG’s Thin E-Strate is more than ten-times thinner than the flexible PP and 20-times thinner than the rigid Arlon 25N, while reducing the whole antenna size more than 15% compared to both of them.

**Figure 4:**
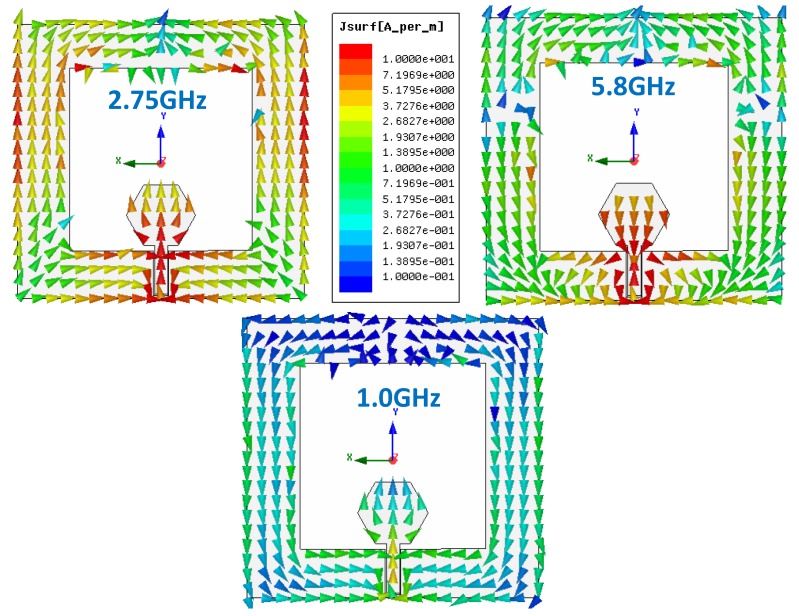
Surface current distribution for the CPW-fed slot monopole at two operative frequencies: 2.75 GHz and 5.8 GHz, and at a not properly matched (non-operative) frequency: 1 GHz.

Figure 4 shows the surface current distribution of the antenna on ENrG’s Thin E-Strate at three different frequencies: Two operative ones (2.75 GHz and 5.8 GHz) and one at which the antenna is not well matched (1 GHz). At 2.75 GHz, high current levels in the vertical lateral sides (referred as metallic strips in the geometry description) can be observed, which agrees with the parametric analysis concerning L and L_w_ influence on the matching at this frequency. Moreover, there is only one null at the upper central part of the geometry, reinforcing the statement with regard to L_L_ influence on the matching. Of course, as expected, there are high currents near the feeding line and the inferior part of the hexagon. At 5.8 GHz, the currents are mainly concentrated in the lower part of the geometry, which agrees with the influence of d (as well as for 2.75 GHz). It is remarkable that there are three nulls: One at the same location as for 2.75 GHz, and two additional ones in the lateral upper sides, which reinforce the idea of W influencing the matching at this frequency and L shifting it downwards. The currents on the hexagon are also remarkable, covering the whole shape with higher value than at the lower frequency, which explains the influence of R_h_ on the shift of the higher frequency band. It has to be kept in mind that this is a slot type antenna, so that the variation on the slot (achieved through the geometry elements variation) is what counts. Finally, at 1 GHz, the current level is low since the antenna is not operative.

### 3.3. Radiation Properties of the Antenna

The radiation properties of the CPW-fed slot monopole antenna have been analyzed in simulation for the three substrates under consideration.

Table 4 indicates the results obtained concerning the peak-realized gain G(dB), the directivity D(dB), and the radiation efficiency η(%) for several frequencies included in both the lower and the upper bands.

It can be observed that the antenna behaves in a very similar way for the three dielectrics. The gain and the directivity are slightly higher for the Arlon 25N and the PP in the upper frequency band. For most WLAN and IoT applications, omnidirectional antennas are preferred so that the directivity is not the parameter to be enhanced. However, the radiation efficiency is the key parameter and the challenging one to be preserved when reducing the antenna size. It can be highlighted that with ENrG’s Thin E-Strate, the radiation efficiency is slightly improved for the lower band compared to the Arlon 25N and for the upper band compared to the PP.

**Figure 5:**
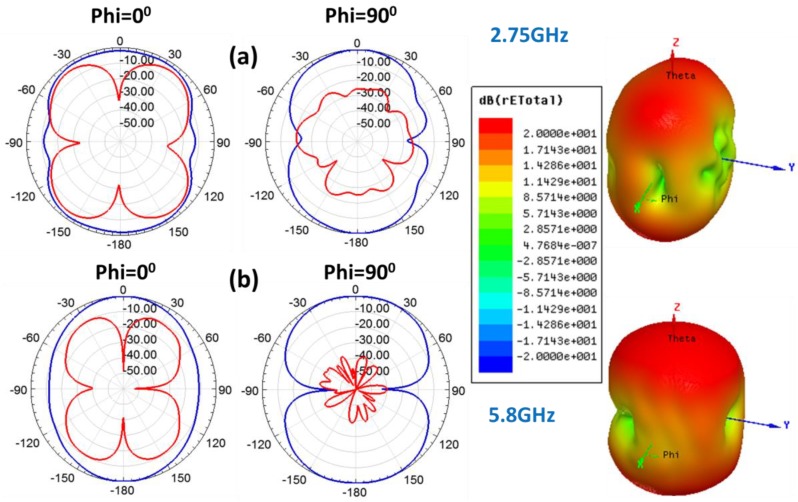
Radiation patterns obtained in simulation for the CPW-fed monopole antenna on ENrG’s Thin E-Strate respectively at 2.75 GHz (**a**) and 5.8 GHz (**b**). The blue traces stand for co-polarization (CP) and the red ones for cross-polarization (XP).

The radiation patterns of the CPW-fed slot monopole antenna on ENrG’s Thin E-Strate have been obtained in simulation for the 2.7GHz and 5.8GHz frequencies. The results in both 3D and the corresponding cuts for Phi = 0^°^ (H-plane) and Phi = 90^°^ (E-plane) are depicted in Figure 5. As expected, the antenna exhibits a monopole-like radiation pattern, quite omnidirectional for the H-plane at both frequency bands.

## 4. Fabricated Antenna on Novel Material

In view of the simulation results, prototypes of the CPW-fed slot monopole based on the novel ceramic ENrG’s Thin E-Strate have been fabricated, aimed at characterizing the antenna based on both return losses and radiation properties.

### 4.1. Fabrication of the Antenna Prototypes

**Figure 6:**
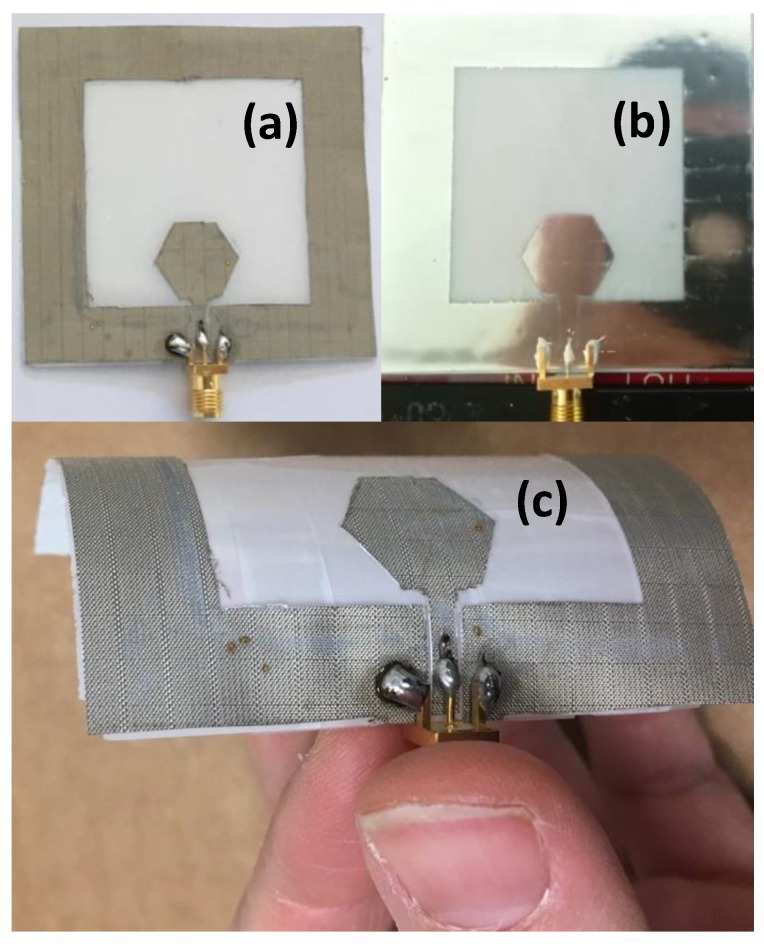
Manufactured prototypes of the antenna on ENrG’s Thin E-Strate using electrotextile flat (**a**); using silver conductive ink (**b**); and using electrotextile bent (**c**).

The conductive parts of the antenna geometry for the first prototype shown in Figure 6a,c, have been realized with Shieldit Super electrotextile, which incorporates a hot-melt adhesive backing, and using laser micromachining with the LPKF Protolaser-S machine.

For the second prototype shown in Figure 6b, a silver-based conductive ink (GenesInk Smart Ink S-CS01130) has been used, printed with the Dimatix DMP-2831 materials printing system using 10 pl cartridges. The ceramic substrate was fixed to the printing area using vacuum for ensuring flatness and avoiding any movement during the printing process. After printing, a 20 min-long thermal process in an oven at 140 °C was needed for curing the ink and achieving good electrical properties. Once the ink was cured, the connector was placed and fixed using Gwent Group C2131014D3 conductive adhesive—a paste developed for screen printing with good adhesion. Once the connector was placed in the adequate position, a thermal process was carried out (120 °C, 10 min).

### 4.2. Experimental Characterization Based on Return Losses

**Figure 7:**
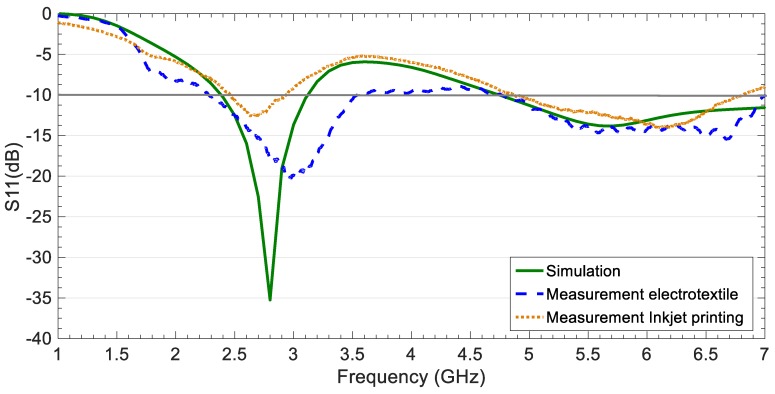
Return losses for the CPW-fed slot monopole antenna on ENrG’s Thin E-Strate in simulation and measurement using electrotextile and inkjet printing as metallization in the fabrication process.

Figure 7 shows the return losses results obtained in simulation and measurement for the fabricated prototypes with electrotextile and inkjet printing. Proper matching was obtained in both cases, getting better agreement compared to simulation for the upper band. The prototypes are suitable for 2.48–2.94 GHz and 4.87–6.82 GHz IoT frequencies, considering the worst case of a first attempt with inkjet printing. Wider bandwidths, 2.39–3.68 GHz and 4.80–6.95 GHz, were obtained using electrotextile, although both techniques could be further improved.

**Figure 8:**
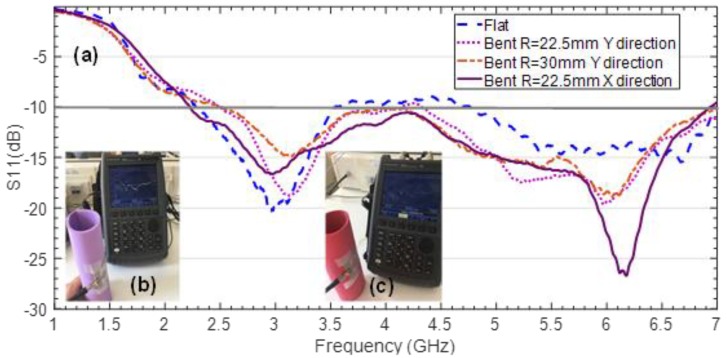
Measurement results concerning the return losses for the CPW-fed slot monopole antenna on ENrG’s Thin E-Strate under flat and bent conditions (**a**). Measurement set-up using a mauve foam cylinder of radius R = 22.5 mm (**b**) and red foam cylinder of radius R = 30 mm (**c**).

The return losses of the antenna were measured under bending conditions. Two foam cylinders with radius R = 22.5 mm and R = 30 mm were used for such purpose. The antenna was bent over the mauve cylinder with radius R = 22.5 mm in both Y and X directions (indicated in Figure 4). For the red cylinder of radius R = 30 mm, only the Y direction is shown, since for the X direction the antenna remains almost flat and, thus, its behavior does not vary significantly compared to the flat condition. The obtained results are shown in Figure 8.

The antenna behavior under bending conditions is quite robust and it seems that the bending conditions should be extreme to significantly perturb its performance in terms of matching. The higher frequency band exhibits greater differences, especially for bending in X direction, which is logical since the wavelength is smaller and, thus, the equivalent electrical bending for a given physical radius is also greater. Nonetheless, the antenna keeps proper matching at the intended frequency bands.

### 4.3. Experimental Characterization Based on Radiation Properties

**Figure 9:**
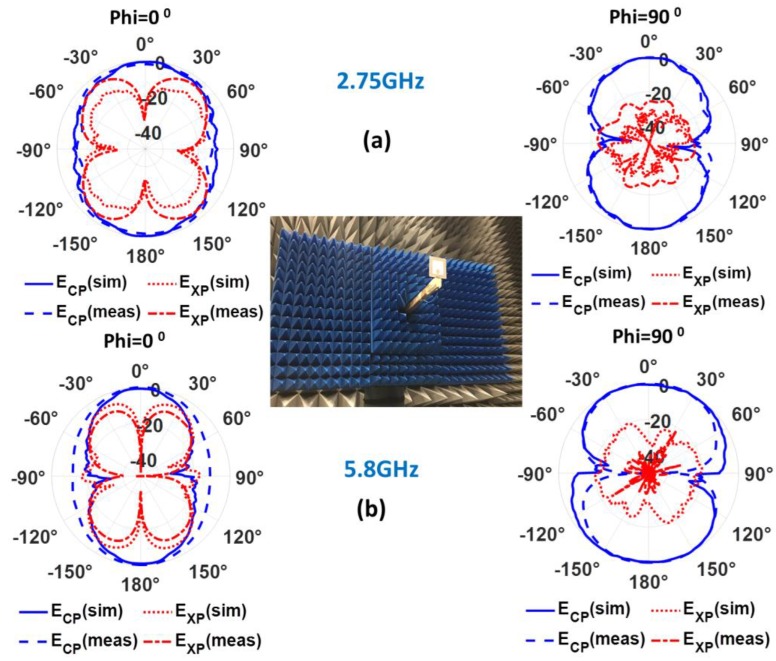
Radiation patterns obtained in measurement for the CPW-fed monopole antenna on ENrG’s Thin E-Strate respectively at 2.75 GHz (**a**) and 5.8GHz (**b**). The blue traces stand for copolarization (CP) and the red ones for crosspolarization (XP).

The radiation pattern cuts of the CPW-fed slot monopole antenna on ENrG’s Thin E-Strate for Phi = 0^°^ and Phi = 90^°^ were measured in an anechoic chamber at 2.75 GHz and 5.8 GHz. The prototype fabricated using electrotextile as metallization was selected for this characterization, since it was the one exhibiting better matching properties in the previous section. The aforementioned measured radiation pattern cuts, along with the ones obtained in simulation, are depicted in Figure 9 for comparison, showing good agreement.

The results obtained in measurement concerning the peak-realized gain G(dB), the directivity D(dB) and the radiation efficiency η (%) at frequencies included in both the lower and the upper bands are indicated in Table 5.

To make a fair comparison with a conventional commercial rigid substrate, a prototype based on ARLON 25N was also fabricated and measured in the same set-up. Compared to the simulation results included in Table 4, it can be mentioned that there is a good agreement. Inter-comparison method was used to obtain the gain values, so that the differences with simulation are attributable to the 0.5dB-1dB tolerance. The directivity was calculated through radiation pattern integration. Small differences with simulation were due to elements used in the measurement set-up to hold the antenna. The radiation efficiency was obtained from both the gain and the directivity, so that the tolerances in both measurements can explain the slight difference with simulations. The variations are similar for both dielectric substrates, flexible ENrG’s Thin E-Strate and rigid conventional ARLON 25N, so that no advantages are attributable to the commercial material in this aspect. Thus, it can be concluded that the flexible ceramic is a really promising material for antenna applications.

## 5. Conclusions

In this contribution, an ultra-thin compact flexible CPW-fed slot monopole antenna, suitable for IoT applications around 2.75 GHz and 5.8 GHz, was designed based on ENrG’s Thin E-Strate^®^.

This novel ceramic dielectric, which electromagnetic characterization was analyzed, provides 20- and 11.25-times thinner antennas compared to Arlon 25N and flexible PP, respectively, while resulting in more than a 15% size reduction.

Antenna prototypes on ENrG’s Thin E-Strate were fabricated using two metallization techniques—electrotextile and inkjet printing—and have been subsequently characterized in terms of return losses with proper results.

The prototype on ENrG’s Thin E-Strate showing better results, was compared with one fabricated on Arlon 25N, in terms of radiation properties obtained through measurement in anechoic chamber. The antenna on ENrG’s Thin E-Strate exhibits excellent radiation performance, while concluding that the conventionally used Arlon 25N has no advantages over the novel Zirconia-based material on these aspects.

The authors are currently involved in improving the fabrication on ENrG’s Thin E-Strate using inkjet printing, not only on silver but also on copper, with promising results.

## Figures and Tables

**Table 1:** Electromagnetic characterization results for the ENrG’s Thin E-Strate with 40 μm thickness at several frequencies using different methods.

Method	Frequency (GHz)	ε_r_	tanδ
Split-post dielectric resonator	1	22.02	0.0011
Split-post dielectric resonator	3	22.19	0.0009
Microstrip T-resonator	4	22	0.001
Split-cylinder resonator	10	22.14	0.0012

**Table 2:** Dimensions of the CPW-fed monopole antenna for different dielectric substrates.

Substrate	Dimensions (mm)								
	W	L	h	W_L_	g	d	R_h_	L_L_	L_W_
Thin E-Strate	66	62	0.04	3	0.26	1.2	8	10	12.0
Arlon 25N	70	69	0.80	4	0.23	1.5	7	13	14.5
polypropylene (PP)	70	69	0.45	4	0.15	1.5	8	12	13.0

**Table 3:** Frequency bands and bandwidths of the CPW-fed monopole antenna for different dielectric substrates.

Substrate	Lower Band				Upper Band			
	Freq (GHz)		BW		Freq (GHz)		BW	
	f_Low_	f_Up_	Total (MHz)	%	f_Low_	f_Up_	Total (MHz)	%
Thin E-Strate	2.383	3.118	735	27	4.754	8.013	3259	51
Arlon 25N	2.490	3.151	661	23	4.808	7.739	2930	47
PP	2.385	3.136	751	27	4.737	6.620	1883	33

**Table 4:** Radiation properties of the CPW-fed monopole antenna obtained in simulation for different dielectric substrates.

Freq. (GHz)	Thin E-Strate			Arlon 25N			PP		
	G (dB)	D (dB)	η (%)	G (dB)	D (dB)	η (%)	G (dB)	D (dB)	η (%)
2.45	4.10	4.46	92	3.87	4.48	87	4.14	4.51	92
2.75	4.52	4.56	99	4.54	4.61	98	4.57	4.61	99
3.00	4.27	4.49	95	4.55	4.78	95	4.54	4.80	94
5.00	4.09	4.47	92	4.33	4.72	91	4.63	4.97	92
5.80	5.19	5.40	95	5.83	6.06	95	5.44	5.75	93
6.50	5.87	6.16	94	6.62	6.88	94	6.36	6.75	91
7.00	6.60	6.91	93	7.49	7.72	95	7.21	7.60	91
7.50	7.21	7.54	93	7.90	8.16	94	7.75	8.17	91

**Table 5:** Radiation properties of the CPW-fed monopole antenna obtained in measurement for the CPW-fed monopole antenna on ENrG’s Thin E-Strate.

Freq. (GHz)	Thin E-Strate			Arlon 25N		
	G (dB)	D (dB)	η (%)	G (dB)	D (dB)	η (%)
2.75	5.17	5.75	88	5.65	5.87	95
3.00	4.58	5.72	77	4.76	5.87	77
5.80	4.30	4.95	86	4.14	4.97	83
6.50	4.63	5.69	78	4.86	6.0	77
